# Bisphenol A-associated alterations in genome-wide DNA methylation and gene expression patterns reveal sequence-dependent and non-monotonic effects in human fetal liver

**DOI:** 10.1093/eep/dvv006

**Published:** 2015-11-05

**Authors:** Christopher Faulk, Jung H. Kim, Tamara R. Jones, Richard C. McEachin, Muna S. Nahar, Dana C. Dolinoy, Maureen A. Sartor

**Affiliations:** ^1^ Department of Environmental Health Sciences, University of Michigan, Ann Arbor, MI, USA 48109;; ^2^ Department of Animal Science, University of Minnesota, Minneapolis, MN, USA 55108;; ^3^ Department of Computational Medicine and Bioinformatics, Medical School, University of Michigan, Ann Arbor, MI, USA 48109;; ^4^ Department of Nutritional Sciences, University of Michigan, Ann Arbor, MI 48109, USA

**Keywords:** bisphenol A, DNA methylation, environmental epigenetics

## Abstract

Bisphenol A (BPA), a high production volume chemical widely used in consumer products, is an endocrine active compound associated with complex epigenetic responses in animal models and humans. Developmental BPA exposure in mice previously revealed widespread changes in the mouse liver methylome. Here, we undertake the first epigenome-wide analysis of the effect of BPA concentration on human fetal liver DNA methylation. Enzymatic enrichment of genomic DNA for high CG density and methylation followed by next-generation sequencing yielded data for positional methylation across the genome. Comparing three groups of BPA-exposed subjects (*n* = 18; 6 per group), high (35.44–96.76 ng/g), low (3.50 to 5.79 ng/g), and non-detect (<0.83 ng/g), revealed regions of altered methylation. Similar numbers of regions of altered methylations were detected in pairwise comparisons; however, their genomic locations were distinct between the non-detect and low or high BPA groups. In general, BPA levels were positively associated with methylation in CpG islands and negatively associated with methylation in CpG shores, shelves, and repetitive regions. DNA methylation at the *SNORD* imprinted cluster (15q11q13) illustrated both linear and non-monotonic associations with BPA levels. Integrated methylation and RNA-sequencing gene expression analysis revealed differential regulation of transcription at low BPA levels, as well as expression changes in RNA for ligand-binding proteins as BPA levels increase. BPA levels in human fetal liver tissue are associated with complex linear and non-monotonic as well as sequence-dependent alterations in DNA methylation. Future longitudinal studies are needed to link these changes with altered health risks.

## Introduction

**Figure 1:**
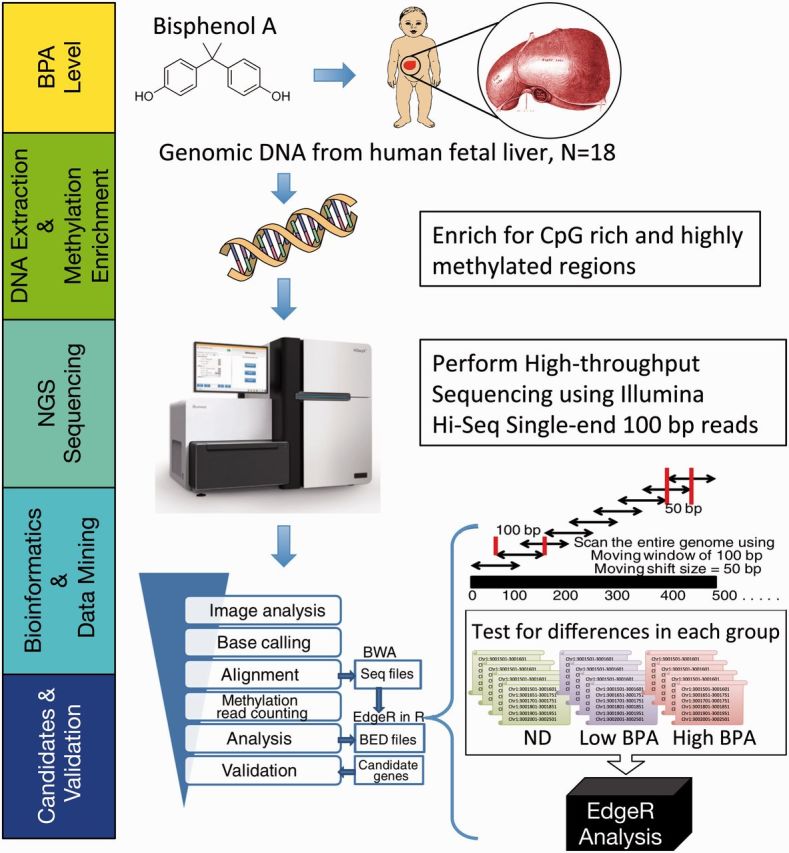
schematic overview of experimental design from BPA quantification in human fetal liver tissue through next-generation sequencing and analysis to identify RAMs. ND, non-detect

Environmental exposures during fetal growth can influence later-in-life health risks, including metabolic and phenotypic outcomes. The developmental origins of health and disease hypothesis posits that chemical and/or nutritional factors during early life result in lasting effects on disease risk, even in the absence of chronic exposure [1, 2]. Growing evidence supports epigenetic inheritance of chromatin marks, such as DNA methylation, as a mechanistic link between fetal exposure and later susceptibility to disease [3, 4]. Adult phenotypic variation deriving from uneven re-establishment of DNA methylation during blastocyst formation is seen across the animal kingdom and can be correlated with environment in the form of stress, chemical exposures, nutrition, maternal behavior, and stochastic effects [5]. Next-generation sequencing enables us to move beyond candidate-gene-based approaches by expanding coverage and sensitivity to detect previously unknown labile regions of the genome that are responsive to early life toxicant exposure. Here, we evaluate bisphenol A (BPA) levels and the DNA methylome in human fetal liver, as an extension of our previously published controlled BPA exposure in mice [6], to uncover BPA’s association with the developing human epigenome (Fig. 1). 

BPA entered commercial production in the 1950s, and by 2008, it had reached global production of 11.5 billion pounds [7], making it one of the highest volume production chemicals in the world. It is used in the manufacture of clear plastics, bottles, can liners, eyeglass lenses, cell phones, thermal receipt paper, and adhesives, among many other products. The safety of BPA has been the subject of long-standing scientific and public debate [8]. A large proportion of the population has detectible levels of BPA in urine [9]. Consumption of canned soup results in >1000% increase in urine BPA [10] and dermal exposure to receipt paper can increase urine concentration from a baseline of 1.8 to 5.8 μg/l [11]. BPA levels in urine have been correlated with obesity in Caucasian children [12]. Pregnant women in southeast Michigan were determined to have between 0.5 μg/l and 22.3 μg/l BPA in circulating blood [13], and our recent study of human fetal livers indicated that most BPA is in the unconjugated form not readily eliminated from the body [14]. These findings strengthen the case that early life development is a particularly crucial window to evaluate BPA’s effects on the epigenome.

Our studies and others have found that developmental exposure to BPA is associated with epigenetic changes in specific tissues at specific genes [15–20] and results in genome-wide changes in liver in rodents [6, 21]. In a cross-sectional study of Egyptian girls, we identified BPA-associated DNA methylation alterations in saliva. Until now, there has been no genome-wide studies of altered epigenetic changes associated with fetal BPA exposure in human tissue. By combining enzymatic methods to enrich genomic DNA for high methylation and high GC content prior to next-generation sequencing, we identified regions of altered methylation (RAMs) between groups of fetal human livers, stratified according to quantified BPA levels. We validated selected top-hit genes to confirm DNA methylation differences and used RNA sequencing to show differential regulation of transcription at low levels of BPA, as well as expression changes in ligand-binding proteins as BPA levels increase. Thus, BPA levels in human fetal liver tissue were associated with complex and sequence-dependent alterations in DNA methylation, as well as by differentially regulating transcription.

## Results

### Analysis Pipeline and Quality Control for Identifying Differential Methylation

We used the MethylPlex-next-generation sequencing (M-NGS) platform to evaluate genome-wide DNA methylation patterns associated with various levels of BPA quantified in human fetal liver samples. This methodology requires minimal DNA input (∼50 ng) and enriches methylated DNA using a cocktail of methylation-dependent restriction enzymes prior to next-generation sequencing (Fig. 1). We confirmed that MethylPlex library reads were enriched in genomic regions containing higher numbers of genes and CpG islands (CGIs). To estimate the false discovery rate (FDR) of the data analysis pipeline (see Methods for details), we employed a sex-based analysis comparing methylation profiles between female and male subjects. Under the conservative assumption that all autosomal regions that pass our filters for significance are false positives, the FDR was estimated to be 11.4% (Supplementary Fig. S1A); however, the actual FDR may be lower, to the extent that true autosomal differences in methylation exist between sexes. The difference in mapped reads on chromosomes X and Y was clearly distinguishable between male and female subjects with minimal background noise observed on chromosome Y from female subjects, confirming the noted sex of each sample (Supplementary Fig. S1B).

### BPA-Associated RAMs

**Figure 2:**
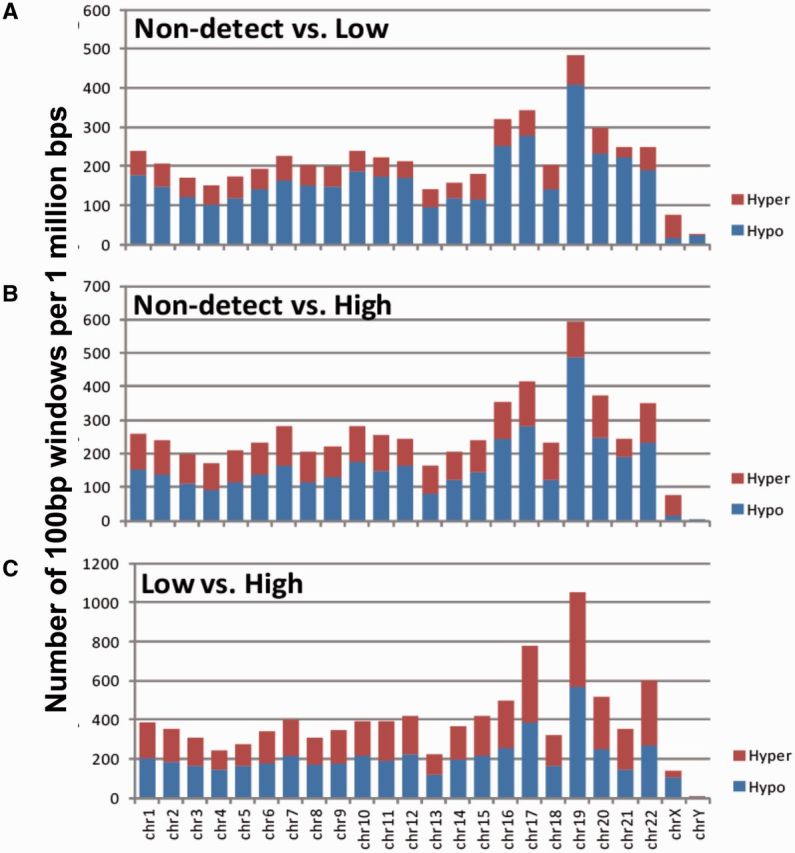
the chromosomal distribution of hyper-methylated (in red) and hypomethylated (in blue) sites as counted by 100 bp sliding windows per 1 million bp, normalized by chromosome length. (**A**) Number of windows in the non-detect vs. low groups are normalized to chromosome length and overall reflect the GC density of each chromosome. The majority of windows were hypo-methylated except for chromosome X. (**B**) Number of windows in the non-detect vs. high BPA groups, similar to (A). (**C**) Number of windows in low vs. high BPA groups shows a balanced number of hyper- to hypo-methylated regions. Overall data were not tested for significance

We identified BPA-associated RAMs using a moving window approach, the *edgeR* Bioconductor package, and post-processing filtering steps, across three BPA categories (non-detect vs. low, non-detect vs. high, and low vs. high), and conducted a refined downstream analysis. As part of data exploration, overall across autosomal chromosomes, we observed a greater number of hypomethylated RAMs with increasing BPA levels, when non-detect subjects were compared with either low or high BPA subjects (Fig. 2A and B). In contrast, when comparing low BPA to high BPA subjects, approximately similar numbers of genomic regions were identified as hyper- and hypo-methylated with increasing BPA levels (Fig. 2C). Chromosome level window counts were not tested for significance. 

**Figure 3:**
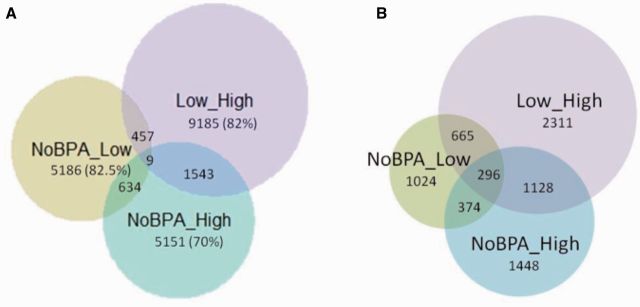
overlap of 100 bp windows with RAMs in non-detect vs. low, non-detect vs. high, and low vs. high BPA groups (**A**) Genome-wide and (**B**) within 5 kb of TSSs of genes

Comparing low BPA subjects with high BPA subjects resulted in the largest number of 100 bp windows with RAMs (11 194). When subjects with non-detectable levels of BPA were compared with those with low and high levels of BPA, similar numbers of 100 bp windows with RAMs were identified (6286 and 7337, respectively), yet these regions were highly mutually exclusive with only 634 windows overlapping (Fig. 3A). A majority of RAMs (19 522 out of a total 24 817 windows) were distinct from one another, suggesting locus-specific and non-monotonic effects of DNA methylation patterns. To identify genic regions associated with RAMs, each candidate 100 bp window was mapped to the nearest gene. In total, 10 005 RAMs were identified within 5 kb of a TSS (Fig. 3B). A total of 296 RAMs were shared across all three comparisons. In contrast to the superset of all RAMs, the TSS-associated RAMs showed greater overlap. 

**Figure 4:**
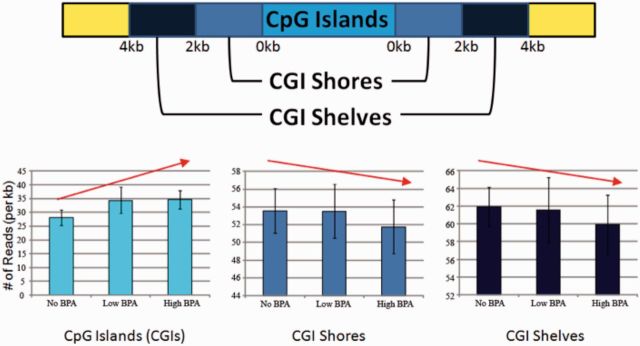
characterization of genome-wide DNA methylation patterns in CGIs, CGI shores, and CGI shelves. Increasing BPA levels were positively associated with DNA methylation in CGIs and negatively associated with DNA methylation in CGI shores and shelves

We also examined the DNA methylation landscape of RAMs around CGIs, CGI shores (0–2 kb from a CGI), and CGI shelves (2–4 kb from a CGI) among the three BPA groups (non-detect, low, and high). We observed a trend of increased methylation in CGIs and decreased methylation in CGI shores and shelves with increasing BPA levels (Fig. 4). These trends did not reach statistical significance. 

**Figure 5:**
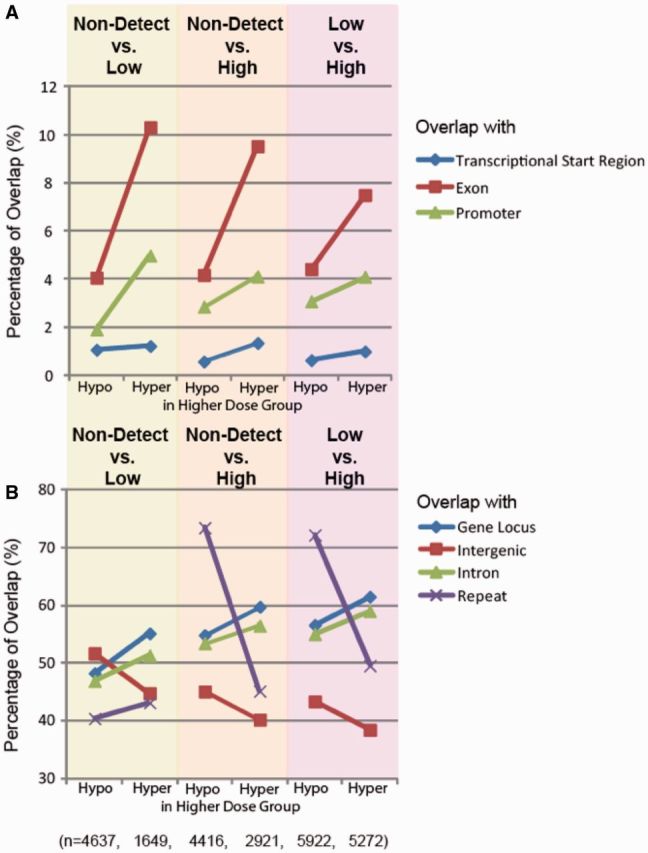
genomic distribution of RAMs. TSRs are defined genomic regions where experimentally verified transcriptional initiation has taken place. Exons are regions of coding sequence. Promoters are defined as −500 to +100 of the TSR. Gene locus encompasses the entirety of the gene, including introns. Intergenic regions are defined as regions outside genic and regulatory regions. Repeats are matched against the UCSC Genome Browser RepeatMasker track. (**A**) A greater percentage of RAMs overlap with exons than TSRs or promoters and have >2-fold higher RAMs overlapping in the relatively higher exposure group, for all comparisons. (**B**) Among gene locus, intergenic regions, introns, and repeats, only hypermethylated RAMs overlapping with repetitive regions are less in the relatively higher exposure groups

In addition, we examined the genomic distribution of RAMs relative to various genomic regions: promoters, or exons, introns, and intergenic regions, or repeat elements. The overview of the statistics of genomic regions overlapping with RAMs is available in Supplementary Table S3. In all three comparison pairs, there were more hypermethylated than hypomethylated regions in the higher dose group in transcriptional start regions (TSRs), exons, and promoters (Fig. 5A). A similar pattern is observed in genomic areas including gene locus, defined as the entirety of the gene transcript, as well as for introns alone (Fig. 5B). However, the reverse pattern is observed in intergenic regions; there were more intergenic hypomethylated regions than hypermethylated in higher dose groups (Fig. 5B). In the non-detect vs. low comparison, around 40.4 of hypo- and 43.2% of hypermethylated regions overlapped with repetitive regions. In both non-detect vs. high and low vs. high comparisons, we observed a striking increase in the percentage of RAMs in repetitive regions among hypomethylated regions in the higher dose group (73.5 and 72.2%, respectively), while the percentage of hypermethylated RAMs in repetitive regions remained similar, at 45.2 and 49.6%, respectively (Fig. 5B). 

### Validation of RAMs

**Figure 6:**
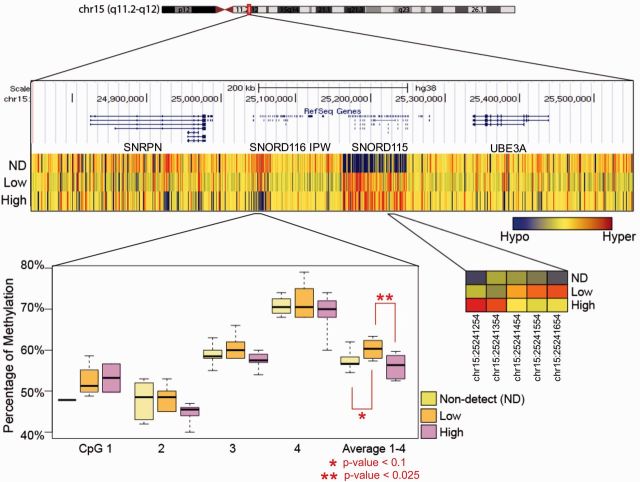
differential methylation at the SNORD gene cluster on chr15. (**A**) Each row in the upper panel represents the average of the six samples in the group. ND, non-detected BPA; low, low BPA level; high, high BPA level. (**B**) Four CpG sites within the SNORD cluster were averaged for validation using pyrosequencing. Exact locations are listed in Supplementary Material and Table S2

To investigate the correlation of BPA level with imprinted gene DNA methylation, we closely examined the snoRNA cluster surrounding *SNORD116*, which is a maternally imprinted locus implicated in Prader–Willi syndrome. In MethylPlex sequencing, *SNORD116* was hypomethylated in the non-detect group and hypermethylated in both the low and high BPA groups (Fig. 6). Validation via pyrosequencing of four CpG sites in the original samples showed linear increased methylation between non-detect and low sample groups (*P* < 0.1) but also detected a non-monotonic response with the average of the low BPA showing higher methylation than the high BPA group (*P* < 0.05). 

A top hit intergenic locus, located on chromosome 7:152888851–152888950, was selected for quantitative DNA methylation validation via EpiTYPER analysis. MethylPlex sequencing resulted in a *P* value < 1.5 × 10^−^^10^ (FDR < 4 × 10^−^^5^) between the low BPA and high BPA groups with the high BPA group displaying less methylation. This pattern was validated by quantitative bisulfite sequencing verifying the non- monotonic DNA methylation (Supplementary Fig. S2).

### RNA-Seq for Differential Expression

Using RNA-seq, we identified seven, one, and three differentially expressed genes in fetal liver for non-detect vs. low, non-detect vs. high, and low vs. high, respectively, using an FDR < 0.10 and 2-fold cutoffs. Although these numbers were small, several high ranked genes were noted to be involved in estrogen processes, e.g. *AHR* [*P* = 0.012 (non-detect vs. low) and *P* = 0.0046 (non-detect vs. high)], *PGR* (*P* value = 0.020), *GPER* (*P* value = 0.025), and *PRL* (*P* = 0.046). Several genes had known BPA-related interactions, e.g. genes in the Wnt signaling pathway were significantly enriched (*WNT2*, *WNT5a*, *WNT7A*, and *WNT11*). Genes related to pregnancy or placental development were also found (*CGA*, *CSH1*, *CSH2*, *INSL4*, *FLT1*, *ADAM12*, *PSG4*, and *PSG5*). The remaining genes came from a variety of categories and include *SERPINE1*, *INDO*, *IGFBP1*, *AREG*, *LOXL1*, *LTBP2*, *FGF9*, *FGF23*, *EGFL6*, *LRP2*, *JAG1*, *NOTCH3*, *THBS2*, and *APOD*.

### Pathway Enrichment Analysis

We performed pathway enrichment analysis with hyper and hypo-methylated RAMs using ChIP-Enrich (http://chip-enrich.med.umich.edu), which assigns peaks to genes based on a chosen method (we used nearest transcription start site, TSS) and tests peaks from ChIP-seq experiments for enrichment of biological pathways, GO terms, and other types of gene sets. Pathway analysis found a strong enrichment of genes involved in metabolism in the low versus high comparison, including regulation of nucleotide metabolic process (*q* value = 0.023), which was enriched among hypermethylated regions, and regulation of metabolic process (*q* value = 0.017), which was enriched among the hypomethylated regions. Genes predicted to be targeted by the aryl-hydrocarbon nuclear receptor translocator were also significantly enriched with hypermethylated regions (*q* value = 0.0098). Among the genes regulated by this transcription factor is the Wilms tumor gene (*WT1*), an imprinted locus. The most consistent enrichment observed across comparisons and both hypo- and hyper-methylation was sets of genes down-regulated in response to estrogen. These gene sets were derived from GEO experiments GSE11324 (3, 6, and 12 h of estrogen treatment), GSE11791, and GSE10879, and all were performed using MCF7 cells (Supplementary Table S4). In particular, breast carcinoma amplified sequence 4 and breast cancer anti-estrogen resistance 3, both involved in the growth and maintenance of breast cancers. In addition, an enrichment analysis based on transcriptomic data from RNA-seq was performed using a logistic regression-based pathway enrichment analysis tool (LRpath) available at http://lrpath.ncibi.org. When enriched concepts among hypomethylated regions and over-expressed genes were compared, the metabolic and catabolic processes as well as cell cycle were significantly enriched in both analyses (*q* < 0.05).

### Integrative Analysis of DNA Methylation and Gene Expression

From the RNA-seq data, we identified 273 (non-detect vs. low), 430 (non-detect vs. high), and 484 (low vs. high) candidate genes, whose expression levels are inversely correlated with methylation levels (Supplementary File S1). When the top enriched biological functions were compared across the three exposure comparison pairs, there were overlapping categories of enrichment shared between the non-detect vs. low and non-detect vs. high groups: across all three comparisons, calcium ion binding genes were enriched (*P* < 5 × 10^−^^4^). Genes in this category included several protocadherins, which are expressed in nervous tissue and includes protocadherin19 which, when mutated, results in an epileptic phenotype in humans [22]. The shared categories between the non-detect vs. high and the low vs. high comparisons included ligand-binding functions, specifically calcium and sulfur compound binding functions (*P* < 4 × 10^−^^4^). Several genes in the sulfur compound binding function have also been shown to interact with BPA according to the Comparative Toxicogenomics Database [23], including lactotransferrin, chemokine ligand 10, follistatin, glypican 4, r-spondin 1, and acyl-coa binding domain containing 5 [24]. Non-detect vs. low or high compared with the low vs. high groups shared only the calcium binding function.

## Discussion

Adaptations of next-generation sequencing to assess DNA methylation and chromatin states are still rapidly evolving, with a variety of currently available approaches. In this study, genomic DNA from BPA-characterized human fetal liver tissue was enriched for methylated regions and high CG density, giving a proxy for differential DNA methylation via regional read-counts. We previously used this approach to show that the mouse liver epigenome responds to BPA exposure at comparable levels found in humans [6]. Our bioinformatics analysis pipeline has been used in mouse [6] and human [25] studies and is validated here by both RNA-seq and quantitative bisulfite sequencing via EpiTyper and pyrosequencing methylation analysis in selected loci. In contrast to the mouse, we find large shifts in RAMs at CGIs. We identified an asymmetric difference in hypo vs. hypermethylated loci in the two exposure groups compared with the non-detect group, while finding similar numbers when comparing low versus high BPA groups. This suggests that BPA exposure biases genes towards hypomethylation in general, as we have previously seen in saliva DNA from a cohort of prepubescent girls [26] and in mouse tail DNA [16]. However, the total number of regions with altered methylation was largest between low and high exposure groups, suggesting that there is a dose-dependent effect. Though our analysis found RAMs between groups to be largely distinct, enrichment analyses pointed to overlapping sets of affected pathways; future studies should be conducted with more granular exposure groups and larger sample sizes to confirm whether the number of genes shifting in methylation concomitant with BPA exposure is nonlinear with dose.

Growing interest in DNA methylation centers on CGI shores and shelves; e.g. in cancers, CGIs have been observed to become hypermethylated, while the surrounding regions tend to become hypomethylated, with an overall loss of signal distinguishing CGIs from their shores and shelves [27]. We add to this data by showing increasing CGI methylation with BPA exposure and decreasing methylation in shores and shelves with BPA. These general results cannot indicate whether a specific locus will have altered expression but would be consistent with reduced expression. Overall, we observed hypomethylation averaged across genic regions; such patterns are also seen in aging cells and tissues [28]. Similarly, long-term epigenetic drift can be impacted by early life exposures and also acts in a locus specific manner [29].

Consistent with the CGI results, RAM location analysis showed higher methylation with higher BPA exposure in the areas most relevant to gene expression: the promoters and TSRs. By raw read count, the highest enrichment by fold-increase of RAMs between all intergroup comparisons (when compared with the genome) occurs in promoter regions; however, the global response of BPA is hypomethylation. The RAM analysis narrows down the location of where the bulk of this hypomethylation is occurring. We observed a decrease in repeat region reads in the high BPA group compared with the non-detect group. Hypomethylation in LINE1 repeat regions with occupational BPA exposure has been documented previously [30]. There was only a small decrease in comparing the low BPA to the non-detect group, but the low vs. high group mirrored the non-detect vs. high group. Given the growing concern over transposon methylation and the importance of silencing [31], BPA's activity in these genomic regions must be carefully examined in depth.

Imprinted genes are an important bellwether of environmentally induced epigenetic change and the SNORD cluster identified here reflects BPA's complex effects. Mouse studies have demonstrated the effect of BPA on imprinted gene methylation through the estrogen receptor signaling pathway [32] and on imprinted gene expression in embryos and placenta [33]. Even low levels of neonatal BPA exposure (2.4 μg/pup) in rats cause persistent hypomethylation in imprinted regions in adult male spermatozoa [34]. Here, despite the SNORD cluster's relatively small size and contiguous maternal imprint, *SNRPN* and adjacent imprinted genes exhibited both linear and non-monotonic responses to BPA exposure. The difference between the MethylPlex and pyrosequencing may be explained by their different regions of assessment: MethylPlex assessed the average of the region, while pyrosequencing probed four specific CpG sites. Interestingly, recent examination of genome-wide DNA methylation patterns in paternal sperm of autism risk children identified differentially methylated genes involved in developmental processes, including many genes in the SNORD family [35].

Pathway analysis was utilized to help understand the likely biological impact of the RAMs, supporting the known estrogenic role of BPA by correlating RAMs with estrogen stimulus response genes. In all comparisons of altered methylation, estrogen responding gene categories were statistically significantly enriched, suggesting that this class of genes is both up- and down-regulated by BPA exposure. Genes targeted by aryl-hydrocarbon nuclear receptor translocator—AHR complex were hypermethylated in low vs. high BPA, which is unsurprising given that BPA is an aryl-hydrocarbon receptor ligand. The RNA-seq GO results taken alone are less clear; however, since methylation does not correspond perfectly to gene expression, integrating the two datasets provides greater insight. Another potential impact may be seen in the detection of pregnancy and placenta-related genes in the RNA-seq GO results, suggesting that differential timing of fetal development may account for the enrichment of these categories across exposure groups. Thus, by integrating the results of methylation data with the RNA-seq data and analyzing GO term enrichment, we found several categories of biological function overlapping between the pairwise comparisons of the non-detect vs. low and non-detect vs. high, especially those relating to gene transcription, suggesting that BPA exposure at both levels has wide-ranging effects on gene expression. In contrast, the genes enriched in the low vs. high group are involved primarily in ligand binding. The only exception to this was in the calcium binding category. Importantly, given the sample source, our low and high groups both represent physiologically relevant concentrations; thus, these results promote the need to examine low-dose BPA exposures in animal studies where the dose ranges may exhibit large gaps, potentially failing to detect discrete but relevant changes.

Ultimately, these results begin to reveal the complexity of BPA's response in human fetal liver at both the DNA methylation and the transcription level. While the global response to BPA exposure is hypomethylation in general, especially in repeats, the CGI hypermethylation effects appear to be both dose and locus dependent. Thus, BPA levels in human fetal liver tissue are associated with complex linear and non-monotonic as well as sequence-dependent alterations in DNA methylation. Furthermore, although the RNA-seq results represent a functional outcome specific to developmental stage (e.g. gestational age) and tissue, the DNA methylation changes may persist across developmental stages and tissues, with only a small subset of the RAMs having a functional consequence for any one time-tissue combination. These limitations can be addressed by future pregnancy studies with larger sample sizes and/or longitudinal studies to link changes in DNA methylation or gene expression with altered health risks and to evaluate target tissue DNA methylation profiles with surrogate DNA, such as saliva or blood, to best inform studies of epigenetic epidemiology, when target tissue DNA is not always readily or ethically available.

## Materials and Methods

### Human Fetal Liver Tissue Samples

Human fetal liver samples (*n* = 50), ranging from gestational days 70 to 120, were procured from the NIH-funded University of Washington Birth Defects Research Laboratory Fetal Biobank (2R24 HD000836-47). As previously described [14, 15, 36], these healthy tissue specimens were collected from voluntary pregnancy terminations after surgery and proper consent from donors and were flash frozen and stored in polycarbonate-free tubing at −80°C until processed for BPA analysis and RNA/DNA extraction (Fig. 1, top box). No identifying clinical data were available on subjects except for gestational age and occasionally sex and race. Thus, samples met the criteria for IRB exemption for human subjects research (UM IRB Exemption: HUM00024929). As described previously, sex was determined from subjects with missing data (*N* = 10) using nested polymerase chain reaction (PCR) assays specific for the Y-chromosome *SRY* and the X-chromosome *ATL1* genes [14].

Total (free plus conjugated) BPA concentrations were measured via high-performance liquid chromatography coupled with an API 2000 electrospray triple-quadrupole mass spectrometer (ESI-MS/MS) by the Kannan Laboratory at the Wadsworth Center (New York State Department of Health) in 0.5 g of *n* = 50 human fetal liver tissue samples and ranged from below the limit of quantification of 0.1 up to 96.8 ng/g [12, 14]. From the wide range of total BPA concentrations quantified in the fetal liver samples, we trichotomized 18 samples with gestational ages ranging from 80 to 115 into non-detect (total BPA concentrations ranging from non-detect to 0.83 ng/g; mean gestational age: 96.2 days), low- (3.5 to 5.79 ng/g; mean gestational age: 105.8 days), and high-BPA (35.44 to 96.76 ng/g; mean gestational age: 103.3 days) exposure groups (*n* = 6 samples per group). The *n* = 18 samples for this analysis were selected from the available set (*n* = 50) based on high DNA and RNA quality, as well as age and gender match per exposure group. Thus, no significant exposure group differences by gestational age were present.

### M-NGS Library Generation

A MethylPlex library synthesis and GC-enrichment kit (Rubicon Genomics Inc., Ann Arbor, MI) was obtained, and the experiments were carried out according to the manufacturer’s protocol (Fig. 1, second box). Methylplex enzymatic enrichment in combination with next-generation sequencing (M-NGS) is capable of identifying RAMs. The method used here was previously described for both human and mouse in Kim et al. [6, 25]. Briefly, a patented cocktail of methylation-sensitive restriction enzymes were used with 50 ng of DNA prior to ligation to universal PCR primer and amplification to create a Methylplex library. A second step enzymatic treatment depleted most non-GC-rich DNA sequences, after which, DNA was re-amplified in a second round of PCR. The adaptor sequences were removed and the product purified prior to incorporation into the Illumina Hi-Seq sequencing platform at the end-repair step of the sample preparation kit according to manufacturer's instructions (Illumina Inc., San Diego, CA). After ligation to Illumina adaptors, the product was run out on a 2% agarose gel with the DNA excised and extracted at the 400 bp position using Qiagen gel extraction kit (Qiagen Inc., Valencia, CA).

### M-NGS Sequencing and Alignment

The purified MethylPlex library was analyzed by Bioanalyzer (Agilent Technologies, San Diego, CA) prior to flow cell generation, where 10 nM of library was used to prepare flowcells with approximately 30 000 clusters per lane. Sequencing was performed by the University of Michigan DNA Sequencing Core on an Illumina HiSeq, and the raw sequencing image data using 100 cycles of single ends were analyzed by the Illumina analysis pipeline (Fig. 1, middle box). An average of 104 million reads per sample (ranging from 78 to 125 million reads) were obtained, where approximately 83.9% of these were mapped uniquely to the human hg19 reference genome using the Burrows-Wheeler Aligner tool (BWA version 0.5.9-r16, parameters: aln -q 6 -t 6) (Supplementary Material and Table S1).

### Differential Methylation Analysis

We applied our tiered-based profiling pipeline developed for mouse BPA exposure studies to identify human locus-specific RAMs (Fig. 1, bottom two boxes), which is described in detail in Kim et al. [6]. Briefly, we scanned the entire genome using a window size of 100 bp with a 50 bp moving shift size, which accounts for over 61 million windows for each sample and obtained the number of mapped reads per 100 bp window per sample. Genomic regions were then filtered to those with at least three samples having a read count greater than 20. The resulting 1.34 million regions were then tested for differential methylation using the *edgeR* Bioconductor package, which we used to test for differences between each pair of exposure groups [37]. The edgeR analysis was run using the glmFit function with tagwise dispersion estimation, which uses a negative binomial model with moderated dispersion estimates, and identified the regions with differential methylation in three different comparisons; the methylation levels from the non-detect group (*n* = 6) against the low exposure group (*n* = 6), non-detect group against high exposure group (*n* = 6), and low exposure group against high exposure group. To minimize the sample-specific methylation variation affecting the results, and because edgeR is sensitive to outliers, we further analyzed the data with filtered RAMs that (i) exhibited methylation change in at least half of the samples per exposure group and (ii) displayed differential methylation either in at least one out of two flanking windows (shift window size is 50 bp) or two 100-bp windows within a 500 bp stretch.

We used the X and Y chromosomes to confirm the sex of each sample and to estimate the overall FDR resulting from using the filtering steps described above. Using the methylation reads mapped to chromosome X and Y, the sex of each human fetal sample (8 males vs. 10 females) was re-confirmed, and the underlying methylation difference among male and female subjects was examined and easily visually distinguished. The above *edgeR* analysis and filtering steps were used to test for male versus female methylation differences. Eight random sets of six males and six females were generated and used for the analysis. Using the conservative assumption that all autosomal methylation differences detected were false positives, we calculated the average percent of significant sites that were autosomal as 11.4% (Supplementary Fig. S1). Thus, we estimate that our FDR is no greater than 11.4% and less to the extent that true autosomal sex-specific differences exist. Three separate BED files containing 6286 unique genomic coordinates from non-detect vs. low, 7337 unique genomic coordinates from non-detect vs. high, and 11 194 unique genomic coordinates from low vs. high analysis were uploaded to Genomatix genome analyzer software (v2. Release 4.7) (https://www.genomatix.de) and mapped to the human genome (NCBI build 37). The classification of regions identifying the overlap with exons, introns, promoters, and intergenic regions, as well as transcription start regions (TSRs) was performed using the RegionMiner workflow. Genomatix defines repeat regions using in-house libraries.

### Gene Set Enrichment Testing

Genome-wide region enrichment of GO terms was performed using the *chipenrich* Bioconductor package (http://chip-enrich.med.umich.edu) [38] with all genomic-regions that passed the filter described above. The nearest TSS locus definition and human reference genome assembly (hg19) were used. The results were visualized using the Reduce and Visualize Gene Ontology (REViGO) web application (http://revigo.irb.hr), which removed redundant GO terms and linked highly similar GO terms with the similarity cutoff value of 0.7 using the *Homo sapiens* database [39].

### Quantitative Methylation Validation

Top candidate regions were selected based on various factors, including *P* values, the number of samples with RAMs, the number of reads, and the methylation status of adjacent regions. Of the two candidate regions selected for validation, the first was in the region located 1.5 kb upstream of *SNORD116-1* in the *SNORD116/SNURF-SNRPN* cluster (chr15:25050592-25050745), and the other was intergenic on chromosome 7 (chr7:152888651-152889150, hg38) proximal to an *AluSg4* transposon exclusive to *Hominidae* between the genes *ACTR3B* and *DPP6*. Genomic DNA (500 ng) from the fetal liver tissue used for M-NGS was bisulfite treated using the EpiTect bisulfite kit (Qiagen Inc., Valencia, CA) to allow for the conversion of unmethylated cytosines to uracil (read as thymine during PCR amplification), whereas the methylated cytosines remain unconverted [40]. Bisulfite-converted DNA (2 µl) was then amplified using Bio-Rad (Model #C1000) thermal cyclers (see Supplementary Table S2 for primer information and PCR conditions) using primers targeting the SNORD gene cluster and the intergenic region on chromosome 7 (chr7:152888651-152889150). PCR products were run on a 1.5% agarose gel to ensure PCR quality, correct product length, and lack of contamination. Following manufacturers’ protocols, amplified SNORD products were analyzed for quantitative DNA methylation levels via the PyroMark Q96 MD Pyrosequencing system (Qiagen, Valencia, CA), and amplified chromosome 7 products were analyzed via the EpiTYPER platform (Sequenom, San Diego, CA). For each primer set, the methylation percentage across individual CpG sites was visualized along with the amplicon average for each sample. The differences in mean amplicon methylation levels in each paired group were tested using a two-tailed *t*-test.

### RNA Sequencing and Analysis

RNA-sequencing was performed on 12 human fetal liver samples (*N* = 4 per group), a subset of the 18 samples profiled with M-NGS, for differential expression analysis using 150 bp single end reads on the Illumina HiSeq analyzed by the Illumina analysis pipeline. Over 438 million reads across 12 samples were obtained, where the average number of reads was 36.5 million per sample. The FastQC tool was used to perform quality control checks on raw data. Because of poor quality (<28) in the second half of the reads, we trimmed reads to 70 bases in length and aligned the reads to the hg19 human reference genome with TopHat, using parameters that accept only the best alignment for reads that align in more than one location and increased searching time to improve sensitivity. After using SamTools to remove duplicate reads to eliminate the effects of PCR duplicates, read counts were generated by CuffDiff software. Differential expression between the three pairwise comparisons (non-detect vs. low, non-detect vs. high, and low vs. high) was tested with *edgeR* Bioconductor package [37], following the developers’ protocol (calcNormFactors, estimateCommonDisp, estimateTagwiseDisp, exactTest, and topTags), using *q* values (Benjamini and Hochberg FDRs) to adjust for multiple testing. Gene set enrichment testing for the RNA-seq data was performed using LRpath (http://lrpath.ncibi.org) [41] against GO and KEGG. Gene set enrichment for the combined Methylplex and RNA-seq datasets were analyzed with GOrilla (http://cbl-gorilla.cs.technion.ac.il) [42] when compared with the complete complement of human protein coding genes (http://genenames.org). GO testing was performed against molecular function at a *P* value threshold of >10^−^^3^ and resulting categories are listed in Supplementary File S1.

## Supplementary Material

Supplementary Data Supplementary Data
