# Supplementary material for: Bisphenol A-associated alterations in genome-wide DNA methylation and gene expression patterns reveal sequence-dependent and non-monotonic effects in human fetal liver
Source: Environ Epigenet. 2015 Nov 5;1(1):dvv006. doi: 10.1093/eep/dvv006 (PMC4922640; doi:10.1093/eep/dvv006)
Supplement: Supplementary Data [file dvv006_supplementary_data.zip › ONES-Human-Manuscript-supp-figures-tables-supp.pdf]

-- Supplementary Material --

Bisphenol A-associated alterations in genome-wide DNA methylation and gene expression patterns reveal sequence-dependent and non-monotonic effects in human fetal liver

Christopher Faulk[1,2§], Jung H. Kim[1,3§], Tamara R. Jones[1], Richard C. McEachin[3], Muna S. Nahar[1], Dana C. Dolinoy[1*], and Maureen A. Sartor[3]

[1] Department of Environmental Health Sciences, University of Michigan, Ann Arbor, Michigan, USA
[2] Department of Animal Science, University of Minnesota, Minneapolis, Minnesota, USA
[3] Department of Computational Medicine and Bioinformatics, Medical School, University of Michigan, Ann Arbor, Michigan, USA
[4] Department of Otolaryngology, Medical School, University of Michigan, Ann Arbor, Michigan, USA
[§] These two authors contributed equally to this work.

Table of Contents

Supplementary Figure S1
Supplementary Figure S2
Supplementary Table S1
Supplementary Table S2
Supplementary Table S3
Supplementary Table S4
Supplementary File S1

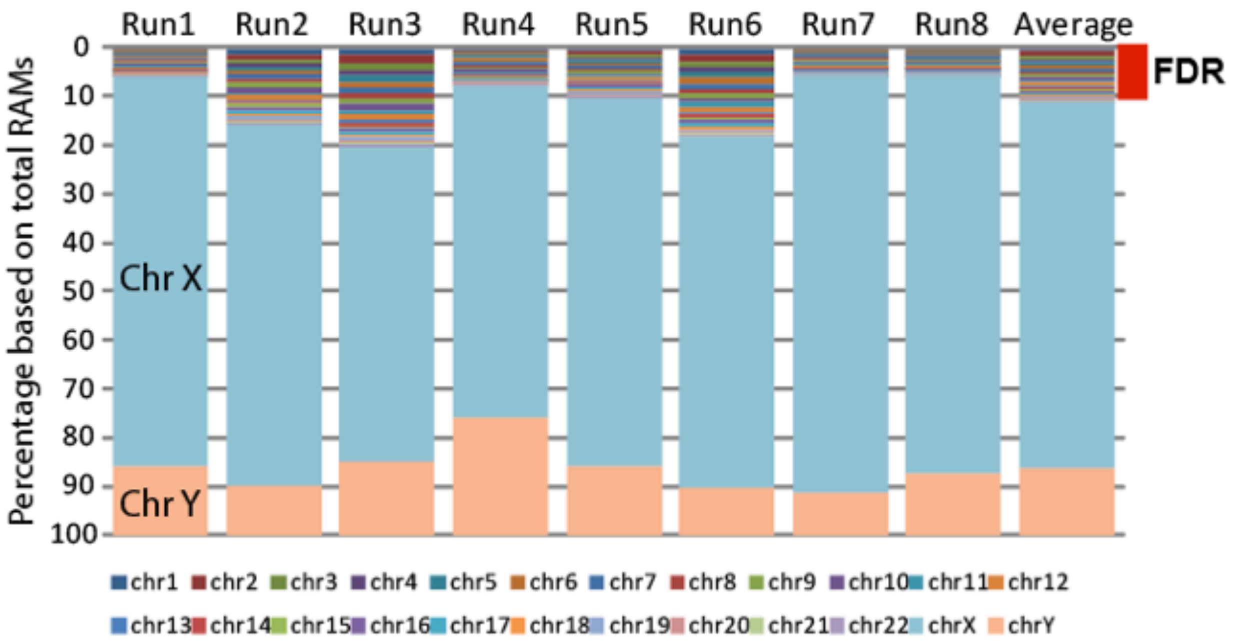

Supplementary Figure S1: Genomic distribution of differentially methylated windows. Comparison of the genomic distribution of RAMs from 6 each of randomly chosen male and female fetal liver samples, repeated 8 times. The majority of RAMs between sexes fall on the sex chromosome as expected. The average false discovery rate (11.4%) was used to as the maximum FDR for the overall analysis in *edgeR*.

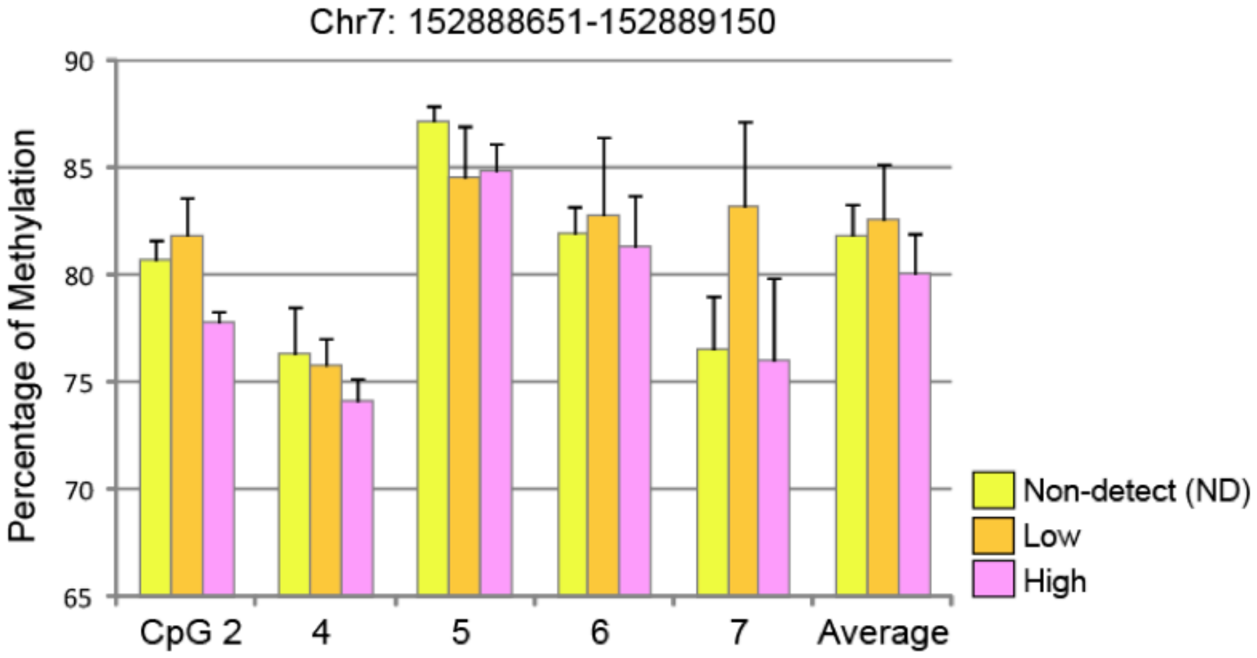

Supplementary Figure S2: EpiTYPER validation on intergenic region Chr7: 152888651-152889150.

Supplementary Table S1: Total Reads Sequenced

Sample ID	Total Sequences	Aligned	Alignment Percentage	Gender	Group
9829	111,733,263	93,269,029	83.5%	M	HIGH
9830	105,038,065	87,180,589	83.0%	M	HIGH
9831	88,895,857	75,420,751	84.8%	F	HIGH
9832	92,124,007	77,931,787	84.6%	M	MEDIUM
9833	100,889,825	85,436,441	84.7%	F	MEDIUM
9834	108,682,540	90,975,939	83.7%	M	LOW
9835	111,062,851	92,922,246	83.7%	F	LOW
9836	100,424,169	83,912,488	83.6%	M	HIGH
9837	78,311,403	65,309,235	83.4%	F	HIGH
9838	84,313,756	70,354,345	83.4%	M	MEDIUM
9839	103,109,938	86,591,994	84.0%	F	MEDIUM
9840	101,554,451	85,420,420	84.1%	M	LOW
9841	102,495,766	86,490,931	84.4%	F	LOW
9842	101,482,571	82,188,294	81.0%	F	LOW
9976	125,109,933	104,429,054	83.5%	F	HIGH
9977	113,959,496	94,233,945	82.7%	M	MEDIUM
9978	118,293,159	100,393,494	84.9%	F	MEDIUM
9979	119,274,331	101,356,486	85.0%	M	LOW

Supplementary Table S2: PCR Primers

Table S2. PCR Primers and Sequences: Primers (5' to 3') and sequences to analyze for DNA methylation quantification via pyrosequencing

Primer/sequence to analyze	SNORD Pyrosequencing Assay chr15:25050592-25050745	Chr7:152888851-152888950 Sequenom Assay (nearest gene ACTR3B)
Forward PCR primer	TATGGTTTGAGGTTAGAGTTT	AGGAAGAGAGaagattaagggaaggaatgtttg
Reverse PCR primer	biotin-ATATATATATCCCTCCCTAAAATAC	CAGTAATACGACTCACTATAGGGAGAAGGCTcactacaacctctaccacccaaat
Sequencing primer	TGATTGAAGATTTTGTTTTTGA	
Sequence to analyze	GTTGTTYGGTTGTTATAGGGT**Y**GGATTTGGAGGTTGTAGATTAT**Y**GTGGTTTTTTGTAGTGTT**Y**GGAAATATATG**Y**GTATTTTAGGGAGGGATATATATAT	
Amplicon length	147	
Temperature	52	
Number of cycles	50	

Sequencing primer amplicon, 4 sites	Chr15:25050622-25050745

Supplementary Table S3: Regions of Altered Methylation Stratified by Exposure

Non-Detect to Low

Type of genomic element	Number of Regions	Percentage of Regions	Percentage in Genome	Enrichment compared to Genome
Exonic, complete	244	3.90%	4.20%	0.9
Exonic, partial	150	2.40%	-	-
Intronic, complete	2826	45.00%	42.90%	1
Intergenic	3066	48.80%	52.90%	0.9
Sum of above	6286	100.00%	-	-
Promoter	205	3.30%	2.50%	1.3

Non-Detect to High

Type of genomic element	Number of Regions	Percentage of Regions	Percentage in Genome	Enrichment compared to Genome
Exonic, complete	306	4.20%	4.20%	1
Exonic, partial	191	2.60%	-	-
Intronic, complete	3703	50.50%	42.90%	1.2
Intergenic	3137	42.80%	52.90%	0.8
Sum of above	7337	100.00%	-	-
Promoter	283	3.90%	2.50%	1.6

Low to High

Type of genomic element	Number of Regions	Percentage of Regions	Percentage in Genome	Enrichment compared to Genome
Exonic, complete	444	4.00%	4.20%	1
Exonic, partial	261	2.30%	-	-
Intronic, complete	5924	52.90%	42.90%	1.2
Intergenic	4565	40.80%	52.90%	0.8
Sum of above	11194	100.00%	-	-
Promoter	449	4.00%	2.50%	1.6

Supplementary Table S4: Gene Set Enrichment by Gene Ontology Stratified by Exposure

See attached file

Supplementary File S1: RNA-seq vs. Methyplex with Gene Ontology Enrichment Analysis

See attached file
